# Atrioventricular Block Subsequent to Intraoperative Device Closure Atrial Septal Defect with Transthoracic Minimal Invasion; A Rare and Serious Complication

**DOI:** 10.1371/journal.pone.0052726

**Published:** 2012-12-28

**Authors:** Qiang Chen, Hua Cao, Gui-Can Zhang, Liang-Wan Chen, Dao-Zhong Chen, Qian-Zhen Li, Zhi-Huang Qiu

**Affiliations:** Department of Cardiovascular Surgery, Union Hospital, Fujian Medical University, Fuzhou, P. R. China; Temple University, United States of America

## Abstract

**Objectives:**

Atrioventricular block (AVB) is a infrequent and serious complication after percutaneous ASD closure. In this study, we report on the incidence of AVB associated with intraoperative device closure of the ASD with transthoracic minimal invasion, and the outcomes of this complication in our center.

**Methods:**

Between May 2006 and January 2011, a total of 213 secundum-type ASD patients were accepted in our hospital for intraoperative and transthoracic device closure with a domestic occluder. All patients were assessed by real-time transthoracic echocardiography (TTE) and electrocardiograph (ECG).

**Results:**

All patients were occluded successfully under this approach. Immediate postprocedure third-degree AVB was observed in two patients. Since heart rates were in the range of about 50 to 55 beats per minute, no intervention was needed except for close observation for one patient. Another patient who recovered sinus rhythm intermittently during the operation was fitted with a temporary pacemaker. Approximately one week following glucocorticoid treatment, the AVB resolved spontaneously in these two patients. Mobitz type II AVB occurred in three patients during the procedure. Two patients developed post-operative cardiac arrest and were rescued successfully with cardiopulmonary resuscitation. One other patient changed to Mobitz type I AVB after three days. During the follow-up period, which ranged from six months to five years, no further occurrence of AVB was found.

**Conclusions:**

Intraoperative and transthoracic device closure of secundum ASDs with domestic occluder resulted in excellent closure rate. AVB is an infrequent but serious complication during and after device closure of a secundum ASD. AVB is a complication that warrants greater attention and long-term follow-up.

## Introduction

Atrial septal defect(ASD) is one of the most common congenital cardiac defects, accounting for approximately 6% to 10% of all congenital cardiac defects. [1], [2] Transcatheter ASD closure has gradually matured as an alternative to surgical closure and has been employed increasingly in recent years, especially in cases of secundum ASD. Cases of secundum ASD have become more amenable to closure since the introduction of the Amplatzer occluder. The major advantage of transcatheter closure is that requires neither a median sternotomy nor cardiopulmonary bypass. In addition, the complication rate for transcatheter ASD closure, is lower than with surgery. [3]–[5] Short- and long-term follow-ups have revealed many of the shortcomings of device closure. [6]–[8] The two-percent incidence of arrhythmia after the device implantation is considerably lower than the thirty-percent incidence reported after surgical closure. [9] The most serious complication experienced by patients has been the occurrence of complete atrioventricular block (AVB). To our knowledge, there are few reports of studies involving large numbers of patients undergoing device closure of ASD. In this article, we reported the incidence of AVB during and after intraoperative and transthoracic device closure ASD using a domestic occluder in our center, as well as the outcome of the complications.

## Materials and Methods

### Device

**Figure 1:**
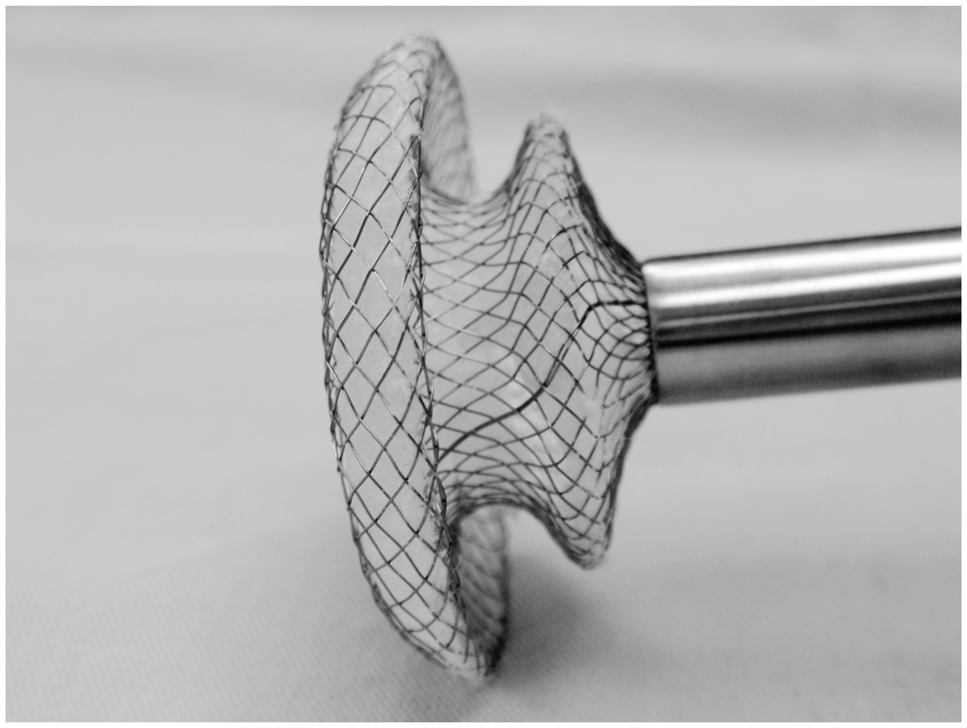
The occlusion devices.

The ASD occluder (Dong Guan Ke Wei Medical Apparatus Co. Ltd, China) was consisted of an occluder made from an alloy of nickel and titanium, a metal sheath, a pushing rod, and a hook. (Fig. 1) The double disc occluder had a loop on the right disc with a 100-cm thread through the loop, facilitating its withdrawal into the sheath, which was 40 cm in length and 4 to 10 mm in diameter sheath. The occluder for each patient was selected in accordance with the transthoracic echocardiography (TTE) result, with a diameter 2 to 6 mm in excess of the maximum defect diameter. The occluder was loaded into the sheath. [10]–[12].

### Protocol

**Figure 2:**
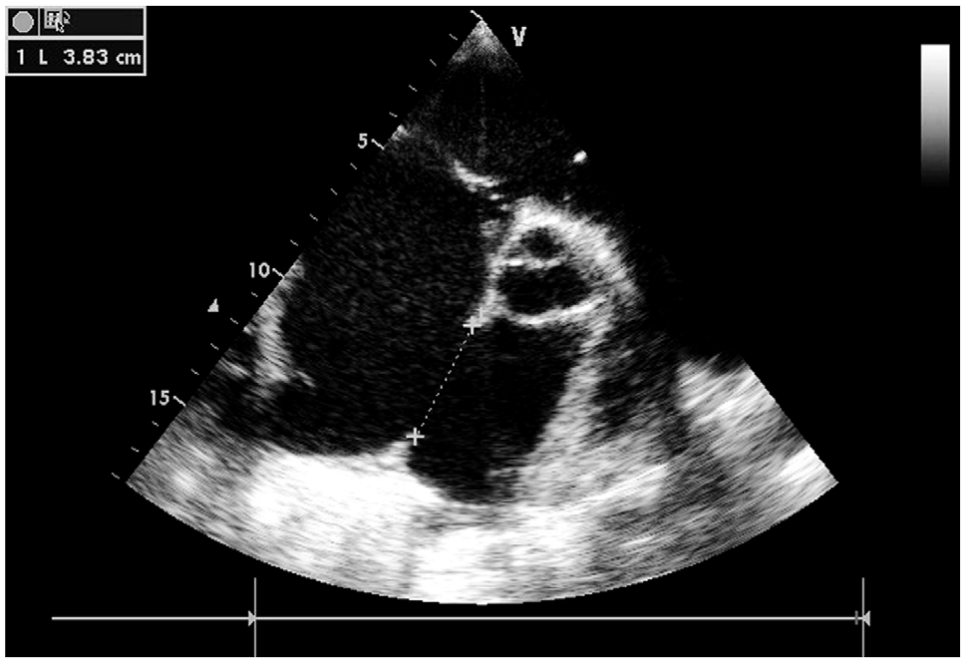
The 38 mm ASD measured by TTE.

The detection of ASD was made by pre-operative TTE, performed initially using standard transthoracic color imaging and Doppler interrogation from subxyphoid, apical, and parasternal views before the closure procedure. During the operation, TTE with a GE vivid 7 multiplane probe (General Electric Company) was used for the accurate determination of ASD location, morphologic features, size (the largest diameter), and visualization of rims. ASD diameter was measured by TTE using two-dimensional imaging and colour flow Doppler on long and short axis views. (Fig. 2) The atrial septal occluder was chosen according to the largest diameter of the ASD, allowed for a margin of 2 to 6 mm in excess of the diameter.

**Figure 3:**
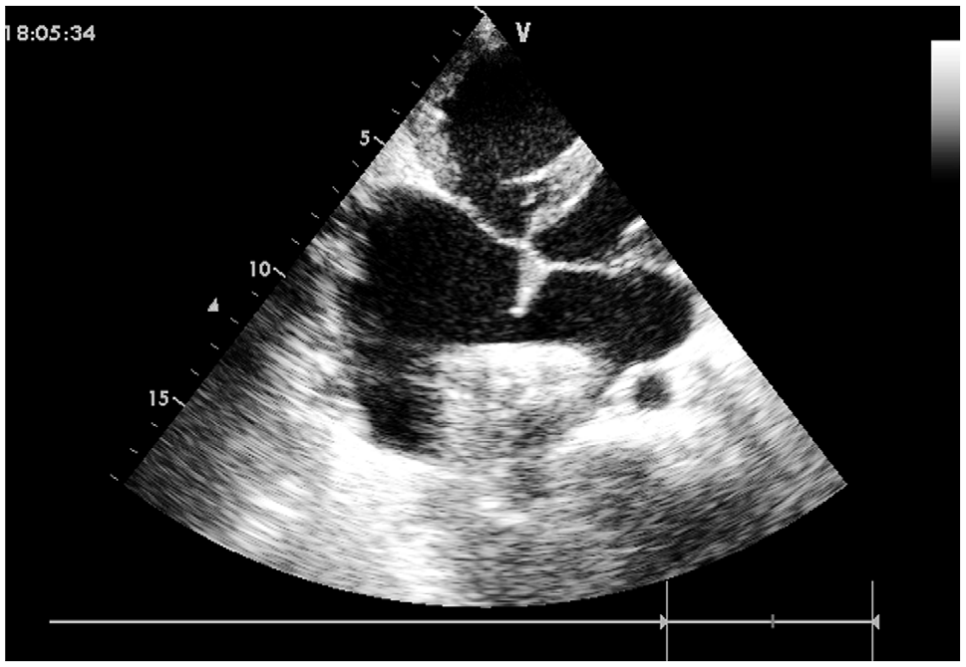
The sheath positioned from the right atrial free wall into the left atrial cavity across the ASD.

**Figure 4:**
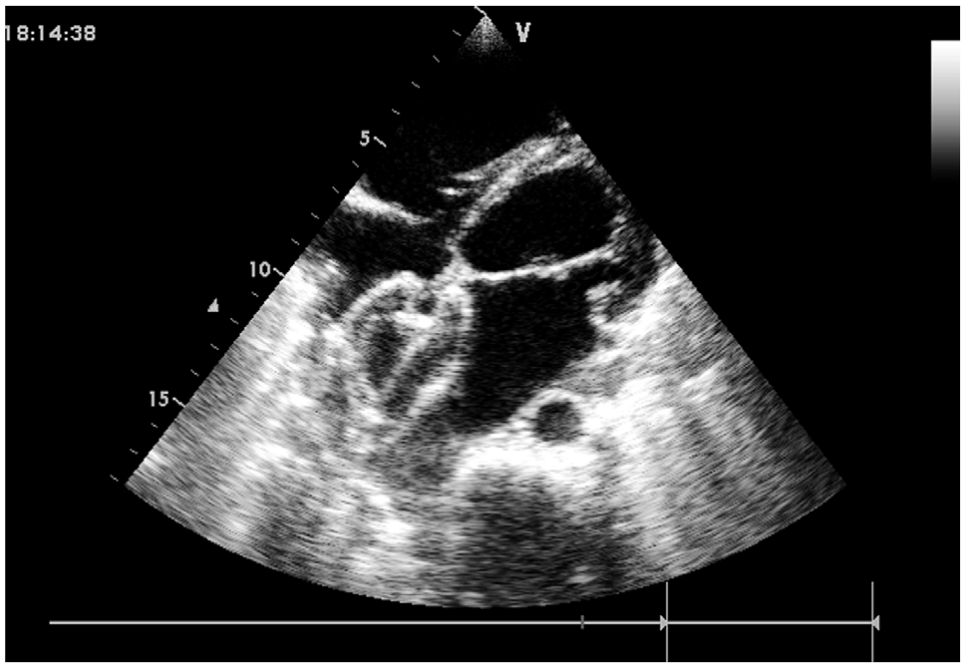
Final image shown after the 44 mm occluder were deployed and the sheath was withdrawn.

Under general anesthesia, each patient was placed in a supine position and draped for exposure of the entire chest, with the right hemithorax elevated approximately 30 degrees. A right anterior submammary minithoracotomy (3–5 cm in length) was made through the fourth intercostal space, and a small rib spreader was used in the manipulation incision to facilitate instrumentation. The pericardium was opened and suspended to expose right atrium. Two parallel sutures of approximately 8 to 16 mm in diameter were placed on the anterolateral right atrium, and heparin (1 mg/kg, IV) was administered to maintain an activated clotting time >250 seconds. After the occluder was drawn into the delivery sheath, a 1-cm incision was opened in the right atrium, and the delivery sheath was inserted. The sheath was advanced under continuous TTE guidance, through the ASD into the left atrium. (Fig. 3) The left disc was deployed first by pushing the rod. The sheath was withdrawn as the left disc was adjusted parallel to the atrial septum, and the right disc was deployed on the other side to occlude the ASD. (Fig. 4) The sheath was moved in a to-and-fro motion to ensure a secure position across the defect. The disc position was assessed using TTE to ensure there was no significantly residual shunt, no distortion of the atrioventricular valve, and no obstruction of the venae cavae or coronary sinus. The thread was cut, and the sheath was withdrawn with the suture tied snugly. The chest was closed using routine procedures, and the patient was instructed to take oral dipyridamole or aspirin for three months as an anticoagulant.

### Participants

In the period between May 2006 to January 2011, 203 patients (98 males and 105 females) with secundum-type ASD were evaluated for intraoperative device closure with our domestic ASD occluder. Those with other coexisting cardiac anomalies were excluded from the study. All patients in the study group received intraoperative device closure. The patients ranged in age from 3 months to 62 years (mean ± standard deviation: 20.0±16.8 years) and weighed from 3.5 to 72 kg (mean: 37.6±20.1 kg). Inclusion criteria for intraoperative device closure were the same as those for surgical or transcatheter closure: hemodynamically significant L-R shunts, and/or significant chamber enlargement, and/or mild-to-moderate pulmonary hypertension despite medical therapy or history of infective endocarditis.

Routine examinations included a standard electrocardiogram, chest X-ray, and blood tests. Arrhythmias were detected in 17 patients before the operation: complete right bundle branch block (1 patient), incomplete right bundle branch block (1 patien), left anterior hemiblock (1 patient), left bundle branch block (2 patients), Mobitz type I AVB (2 patients), sinus bradycardia (5 patients), premature atrial beats (3 patients), and atrial fibrillation (2 patients)).

## **Ethics**

The present study was approved by the ethics committee of our university and was conducted according to the tenets of the Declaration of Helsinki. Written informed consent was obtained from the patients or their parents.

### Statistical Analysis

Continuous data were presented as mean± standard deviation and range.

## Results

Delivery of the occluder was successful in all patients. The size of the ASD as measured by TTE ranged from 5 to 44 mm (mean 20.8±10.8 mm). The size of the implanted occluder ranged from 6 to 48 mm (mean 23.8±11.2 mm). Immediate post-procedure third-degree AVB was observed in two patients. Since their heart rates ranged from about 50–55 beats per minute, no intervention was needed except for close observation of one patient. Another patient recovered sinus rhythm intermittently during the operation and had a temporary pacemaker inserted. The AVB resolved spontaneously in these two patients after about one week of glucocorticoid treatment. Mobitz type II AVB occurred in three patients during the procedure. Two patients developed post-operative cardiac arrest and were successfully resuscitated. One other patient changed to Mobitz type I AVB after three days. New minor transient arrhythmia complications-temporary sinus bradycardia, premature atrial beats, and sinus tachycardia-developed in 32 patients in the course of the device deployment. These conditions were treated easily with drugs or resolved spontaneously. Immediate post-procedural Mobitz type I AVB was observed in one patient, requiring only close observation.

In those patients who had a successful attempt, the total follow-up period ranged from six months to five years. Out-patient follow-up was by functional, echocardiographic, and ECG assessment. There were no instances of progressive arrhythmia. To date, none of the patients in the study has developed complete AVB.

## Discussion

Atrial septal defect is one of the most common congenital cardiac defects. Elective open-heart repair with midline sternotomy and cardiopulmonary bypass has been considered the gold standard for ASD closure. With the development of various devices, percutaneous transcatheter closure of ASD gradually become a viable alternative procedure for selected patients. The safety and feasibility of transcatheter ASD closure have been confirmed by the use of the Amplatzer septal occlude (ASO) in many centers, and only limited complications have been reported in various studies. [13]–[16] We used a hybrid method, which included an intraoperative device and a minimally invasive procedure to close the ASD. Our method was easy to learn and cost-acceptable in the Third World nations. Similar to previous reports on transcatheter ASD closure, we also achieved high technical success and good therapeutic outcomes. Although there is little published data on arrhythmia analysis following transcatheter device closure of secundum ASD, complete heart block is a rare complication of device closure. In this study, we report our experience with intraoperative device closure of ASD and the incidence of AVB associated with the procedure.

Complete AVB associated with ASD device closure has been described sporadically in several studies and case reports, in most of which the AVB was transient and recovered in a short period of time. Lin et al. [17] report the case of a nine-year-old boy who presented with complete AVB after undergoing percutaneous closure of a large secundum ASD with an ASO. The patient was treated with oral prednisolone, and the atrioventricular conduction improved to second-degree Mobitz type I block on post-procedure day 24 and first-degree block on day 35. Nehgme et al. [18] report the case of a six-year-old girl who, four years after ASD closure with an ASO, presented with progression of AVB to symptomatic complete heart block requiring implantation of a permanent pacemaker. Johnson et al. [19] reported that 2 of 610 patients with different device closures suffered clinically significant heart block post-procedure. One patient had a history of congenital heart disease. The other had evidence of second-degree heart block as detected by pre-procedure ECG. They recommended patients with clinically significant second-degree heart block who were considering percutaneous device closure should be advised of the potential risk for developing a more severe block after device closure. Suda et al. [20] reported that 10 patients (6.2%) presented with new-onset AVB or showed aggravation of preexisting AVB. In their series, all AVBs resolved or improved spontaneously, with no recurrence at mid-term follow-up. ASD closure using a large ASO can be associated with the development of AVB and warrants a closer follow-up. In our study, the incidence of complete AVB was about 1%.

The exact mechanism underlying AVB following ASD closure remains speculative. We can hypothesize that the possible mechanism of AVB is an inflammatory response and subsequent edema as a correlative result of mechanical rubbing of the occluder against the proximal conduction system. The margins of the secundum atrial septal defect include Bachmann’s bundle, the primary path for electrical conduction from the sinoatrial node to the atrioventricular node. The atrioventricular conduction bundle, which emerges from Bachmann’s bundle in the atrioventricular node area to conduct electrical impulses to the Purkinje fibers, is especially prone to damage. Injury to the conduction system that results from mechanical trauma/compression by the delivery system or by the device to cause acute intra-procedural complete AVB has a reasonable probability of early resolution. Adequacy of the inferior rim is also assumed to be necessary to avoid this complication. Al-Anani et al. [21] emphasized that the risk is more pronounced in patients with deficient posterior-inferior rims (<5 mm). Assessment of the real spatial relationship between the occluder and the AV node using TTE, may give us more insight on this issue. Some cases of early complete AVB have been reported to resolve spontaneously or after temporary corticosteroid therapy or device removal [17], [20], [21]. We observed immediate post-procedural complete AVB in two patients. Since their heart rates were in the range of 50 to 55 beats per minute, no intervention was needed except for the close observation of one patient. This patient maintained stable hemodynamics and sufficient exercise tolerance post-operation despite the low heart rate. Another patient recovered sinus rhythm intermittently during the operation, with lengthening of the PR interval and sinus cardiac arrest after device placement. This individual was considered at risk for developing subsequent malignant events, so a temporary pacemaker was inserted. Two patients required glucocorticoid treatment for about one week, after which the AVB resolved spontaneously. Whether or not these were spontaneous or corticosteroid-enhanced recovery remained uncertain. Some authors have suggested that a course of steroids may be useful in reversing AVB. But use of corticosteroids to enhance AV conduction recovery is not supported by experimental data or controlled studies, the effectiveness of these drugs has yet to be determined [17], [20], [21]. Al-Anani et al. [21] reported that two patients with complete AVB were ultimately sent for surgical removal of their devices with complete resolution of their atrioventricular conduction abnormalities. However, it is uncertain if early device removal would have prevented this complication.

It is intuitive to surmise that the AVB occurring immediately after device occlusion may result directly from mechanical trauma/compression. Careful manipulation of devices while correcting the defect and during release is important. Compared with transcatheter closure of ASD, our approach offers a clear operative field and allows cardiac surgeons to use traditional surgical techniques more effectively. This approach also provides a perpendicular angle to the atrial septum, allowing the sheath to be guided through the ASD and the occluder to be deployed into the defect with greater ease. In our experience, the chance of injury to the conduction system from mechanical trauma/compression by the delivery system or device was small.

Suda et al. [20] concluded that shunt diameter and device size were the only determinant factors for AVB. Device larger than 19 mm in diameter were used in 90% (9 of 10) of the patients who developed AVB, compared with only 49% of those who did not develop AVB. Indeed, the size of the device can be a predisposing factor of AVB after ASD closure. In our approach, the size and position of the device was verified carefully using TTE before and after deployment. Although we agree that the use of transesophageal echocardiography (TEE) to assess the size, number, and position of the defects is prevalent, in our opinion, traditional TTE performed by an experienced operator is still a reliable method for measuring ASD diameters and circumferential margins. A large occluder might compress the AV node, producing edema and inflammation of the adjacent cardiac tissue secondary to friction from the occluder. Precise measurement of the ASD so as to avoid placing an oversized device is important to minimize the risk of AVB. The device selected should be 2 to 6 mm larger than the ASD, as measured by TTE. From the little evidence that we have to date, the use of an oversized device should be avoided. The first complete AVB case in our study was in a 54-year old individual with an ASD diameter of 40 mm and a occluder size of 44 mm, in whom sinus bradycardia emerged pre-operation. The second was in a 48-year old individual with an ASD diameter of 38 mm and a occluder size of 42 mm, in whom left anterior hemiblock occurred pre-operation. Our results agree with that large occluder size is a risk factor for the occurrence of the AVB.

It is well known that ASDs may be associated with subclinical ECG abnormalities, including sinus node dysfunction, conduction delay, and AVB. Preexisting sinus node dysfunction or AV conduction disturbance should not be ignored, especially in the case of large ASDs requiring large devices. Twelve-lead ECG periodically and 24-h Holter recordings are necessary later on. In our series, Mobitz type II AVB occurred in three patients during the procedure. Two patients developed post-operative cardiac arrest and were rescued by cardiopulmonary resuscitation. These two patients had pre-operative left bundle branch block or Mobitz type I AVB and were more than 50 years of age. After occlusion, transient and significant left-ventricular volume overloaded and a slow heart rate may result in cardiac arrest. After treatment of with dopamine and furosemide for several days, both patients recovered well. Another patient changed to Mobitz type I AVB after three days. All three patients were treated with a glucocorticoid for three days. It has been suggested that older patients might be at higher risk than young patients for developing AVB.

It remained unclear why certain patients developed complete AVB and others do not, and why it occured early in some patients and much later in others. There are few published reports dealing with late-onset complete AVB. Al-Anani et al. [21] emphasized several important points. This complication can present at anytime from immediately after cardiac catheterization to even 48 hours later. Moreover, these changes can be subclinical and can progress either slowly or rapidly to complete AVB. Anti-inflammatory agents may or may not be beneficial. Nehgme et al. [18] reported the case of a 6-year-old girl who presented with progression of first-degree AVB to symptomatic complete AVB that required implantation of a permanent pacemaker four years after ASD closure with an ASO. We can only speculate that the injury to the conduction system was the result of persistent trauma, ischemia, fibrosis, or progressive scarring caused by the ASO on the AV nodal region. Although we had not experienced this type of complication, it seems that complete AVB may appear several months or years following device closure of the ASD. Patients and their parents should be informed the risk of sudden, potentially life-threatening late-onset complete AVB. It implies that long-term follow-up, perhaps life-long, should be mandatory. The chance of the delayed onset of complete AVB is the same for transcatheter and intraoperative device closure. More attention should be given to the routine follow-up.

It is a difficult to know how to avoid AVB. We recommended abandoning the procedure once complete AVB occurs. Some authors have suggested that a course of steroids may be useful in reversing AVB, but the effectiveness of these drugs has yet to be determined. We used this approach successfully in our AVB cases. These is still controversy about whether and when to implant a permanent pacemaker for patients who developed complete AVB after device closure of the ASD. Likewise, the indication for and timing of device retrieval are not available in the literature. It is our opinion that surgical closure remains the treatment of choice for those patients who present complete AVB after device closure.

This study presents a single-center experience involving a small number of patients. It focuses on complete AVB occurring in the course of intraoperative device closure of secundum-type ASD. It is not representative of the entire ASD device experience, which includes many older and larger patients and smaller defects. Our study was conducted in low-income countries with limited health care resources. Although the techniques of intraoperative device closure of the ASD appear to be safe in the short-term follow-up, it is not known whether they are safe over the long term. Given these circumstances, longer follow-ups are needed in future research.

In conclusion, our study demonstrates that intraoperative device closure of secundum ASDs is feasible and safe according to short-term follow-up. Complete AVB was a serious complication during and after ASD device closure in a very small proportions of patients. No risk factor for complete AVB was clearly identified. More attention must be paid to this complication in the future, and long-term follow-up is essential for patients who have had the device implanted.
